# Docosahexaenoic acid prevents dendritic cell maturation and in vitro and in vivo expression of the IL-12 cytokine family

**DOI:** 10.1186/1476-511X-9-12

**Published:** 2010-02-01

**Authors:** Weimin Kong, Jui-Hung Yen, Evros Vassiliou, Sabina Adhikary, Miguel G Toscano, Doina Ganea

**Affiliations:** 1 Department of Physiology, Temple University School of Medicine, 3500 North Broad Street, Philadelphia, Pennsylvania 19140, USA; 2 Department of Microbiology and Immunology, Temple University School of Medicine, 3500 North Broad Street, Philadelphia, Pennsylvania 19140, USA; 3 Department of Biological Sciences, Kean University, 1000 Morris Avenue, Union, New Jersey 07083, USA

## Abstract

**Background:**

Acute and chronic inflammation play essential roles in inflammatory/autoimmune conditions. Protective anti-inflammatory effects of the n-3 fatty acids docosahexaenoic acid (DHA) and eicosapentaenoic acid (EPA) were reported in animal models of colitis, sepsis, and stroke. Since dendritic cells (DC) represent the essential cellular link between innate and adaptive immunity and have a prominent role in tolerance for self-antigens, we sought to investigate the impact of DHA on DC maturation and proinflammatory cytokine production.

**Methods:**

Murine bone marrow-derived DC were treated with DHA and stimulated with various toll-like receptor (TLR) ligands. Flow cytometry was used to determine the levels of surface maturation markers and endocytic activity. Cytokine expression and secretion were measured by real-time RT-PCR and ELISA assays. PPARγ and NFκB activity in nuclear extracts were determined by binding to specific oligonucleotide sequences using ELISA-based assays. In vivo effects of DHA were assessed in splenic DC from LPS-inoculated mice maintained on a DHA-enriched diet.

**Results:**

DHA maintained the immature phenotype in bone marrow-derived DC by preventing the upregulation of MHCII and costimulatory molecules (CD40, CD80 and CD86) and maintaining high levels of endocytic activity. DHA inhibited the production of pro-inflammatory cytokines, including the IL-12 cytokine family (IL-12p70, IL-23, and IL-27), from DC stimulated with TLR2, 3, 4, and 9 ligands. DHA inhibition of IL-12 expression was mediated through activation of PPARγ and inhibition of NFκBp65 nuclear translocation. DHA exerted a similar inhibitory effect on IL-12 and IL-23 expression in vivo in LPS-inoculated mice maintained on a DHA-enriched diet.

**Conclusions:**

Exposure of bone marrow-derived DC to DHA resulted in the maintenance of an immature phenotype and drastic reduction in proinflammatory cytokine release. DHA inhibited the expression and secretion of the IL-12 cytokine family members (IL-12p70, IL-23 and IL-27), which play essential roles in the differentiation of the proinflammatory Th1/Th17 effector cells. The effect of DHA on IL-12 expression was mediated through activation of PPARγ and inhibition of NFκB. Inhibition of IL-12 and IL-23 expression was also evident in splenic DC from mice fed a DHA-enriched diet, suggesting that dietary DHA acts as an anti-inflammatory agent in vivo.

## Background

In contrast to n-6 polyunsaturated fatty acid (PUFAs) such as arachidonic acid (AA) which mediate predominantly proinflammatory effects, the n-3 PUFAs are mostly anti-inflammatory. The anti-inflammatory effects of docosahexaenoic acid (DHA) and eicosapentaenoic acid (EPA) have been attributed initially to the inhibition of COX mediated metabolism of AA. More recently, DHA and EPA were shown to be the precursors of potent anti-inflammatory derivatives such as resolvins and protectins [reviewed in [1-8]].

DHA and EPA are obtained primarily from diet but are also synthesized endogenously in the liver from α-linoleic acid through a series of desaturation and elongation reactions. Circulating free fatty acids enter cells, followed mostly by esterification via acyl-CoA transferase into membrane-residing 2-lysophospholipids [4,8]. It has been proposed that following proinflammatory signaling, DHA is cleaved from membrane phospholipids by a calcium-independent phospholipase and processed by 15-lipoxygenases (15-LOX) into protectins and D-series resolvins [reviewed in [4,9]]. In addition, circulating n-3 fatty acids also represent an important source of anti-inflammatory mediators, as reported in a recent study showing rapid accumulation of circulating DHA and EPA in peritoneal inflammatory exudates [10].

Most of the studies related to the anti-inflammatory activities of n-3 fatty acid derivatives, *i.e*. resolvins and protectins, have been focused on their effect in resolving inflammation mostly through reduction in neutrophil trafficking and upregulation of macrophage-mediated removal of apoptotic cells [reviewed in [6,9,11,12]]. Few studies reported effects of n-3 fatty acids on dendritic cells (DC) [13-15]. DC represent the essential cellular link between innate and adaptive immunity. Resident DC activated by pathogens exhibit high endocytic capacity. Following TLR signaling, conventional DC upregulate the expression of MHC and costimulatory molecules, produce proinflammatory cytokines and chemokines, and undergo a change in chemokine receptors. As a result, activated DC migrate to lymph nodes where they stimulate naïve cognate T cells. In addition, DC can also function as inducers and maintainers of tolerance to self antigens following uptake of apoptotic cells and induction of anergic or regulatory T cells [reviewed in [16,17]].

Through direct release of proinflammatory factors and induction of proinflammatory Th1/Th17 effectors, DC play an essential role in inflammatory/autoimmune conditions. Therefore, the identification and characterization of endogenous and/or exogenous anti-inflammatory agents such as the n-3 fatty acids and their derivatives represents an active field of research with significant therapeutic potential. In the present study we determined that exposure of conventional (myeloid) DC to DHA contributes to the maintenance of an immature phenotype and prevents expression of proinflammatory cytokines through PPARγ activation and inhibition of NFκB nuclear translocation. We also observed a similar reduction of IL-12 family cytokines in vivo in splenic DC obtained from mice maintained on a DHA-enriched diet.

## Materials and methods

### Mice

B10.A (6 to 10 weeks old) and C56BL/6 (4 weeks old) mice were purchased from Jackson Laboratory (Bar Harbor, ME). Mice were handled and housed in accordance with the guidelines of the Temple University Animal Care and Use Committee.

### Reagents

Lipopolysaccharide (LPS) (*Escherichia coli *O26:B6), polyinosinic-polycytidylic acid (PolyI:C), peptidoglycan (PGN), fluorescein isothiocyanate (FITC)-conjugated dextran and streptavidin-peroxidase were purchased from Sigma-Aldrich (St. Louis, MO). CD11c MicroBeads were purchased from Miltenyi Biotec (Bergish-Gladbach, Germany). CpG ODN 1826 was purchased from InvivoGen (San Diego, CA). Recombinant murine GM-CSF, IL-12, TNFα, IL-6 and CCL-4 were purchased from Peprotech Inc (Rocky Hill, NJ). The ELISA kit for murine IL-27p28 was purchased from R&D Systems (Minneapolis, MN). DHA, GW9662, Rosiglitazone (Rosi) and peroxisome proliferator activated receptor γ (PPARγ) transcription factor assay kit were purchased from Cayman Chemical (Ann Arbor, MI). Capture and biotinylated anti-mouse IL-23 antibody were purchased from eBioscience (San Diego, CA). FITC-conjugated anti-mouse CD80, CD86, CD40, MHCII; recombinant mouse IL-10; capture and biotinylated anti-mouse IL-12/p70, IL-6, TNFα, IL-10 and CCL-4; and TMB Substrate Reagent Set were purchased from BD PharMingen (San Diego, CA). Nuclear extract kit and TransAm NFκB p65 transcription factor assay kit were purchased from Active Motif North America (Carlsbad, CA). Anti-IκBα mouse mAb were purchased from Cell Signaling Technology, Inc (Danvers, MA). Anti-GAPDH rabbit mAb were purchased from Fitzgerald Industries International (Concord, MA). Goat anti-rabbit and goat anti-mouse antibodies conjugated to infrared dye were purchased from LI-COR (Lincoln, NE).

### Generation and purification of DC from bone marrow

DC were generated from bone marrow. Briefly, femur and tibiae were removed from 6- to 8-wk-old male B10.A mice. Both ends of the bones were cut open and bone marrow cells were flushed out and washed with ice-cold RPMI 1640 medium (Invitrogen Life Technologies Research Laboratory). 2 × 10^6 ^bone-marrow cells were cultured in 100 mm petri dishes containing 10 ml RPMI 1640 medium supplemented with 10% heat-inactivated FBS (Atlanta Biologicals, Norcross, GA), 2 mM L-glutamine, and 20 ng/ml recombinant GM-CSF. After three days, another 10 ml of complete medium containing GM-CSF was added to each dish. On day 7 the non-adherent cells were harvested and purified by immunomagnetic sorting with anti-CD11c-coated magnetic beads using the autoMACS system according to the manufacturer's instructions (Miltenyi Biotec). The purity of the sorted cells was determined by FACS analysis (>96% for CD11c+ cells).

### FACS Analysis

Cells were subjected to FACS analysis in a 3-color FACSCalibur (BD Biosciences, Mountain View, CA). Data were collected for 10,000 cells and analyzed using Cellquest software from BD Biosciences (San Jose, CA). DC were cultured in 12-well culture plates (1 × 10^6^/ml) and pretreated with 50 μM of DHA for 24 h, followed by LPS (0.1 μg/ml) for an additional 24 h. DC were collected, washed with PBS and incubated for 30 minutes at 4°C with anti-CD80 FITC, anti-CD86 FITC, anti-CD40 FITC, anti-I-E^k ^FITC. The specificity of the primary Abs was established with appropriate isotype-matched controls.

### Endocytosis

Endocytosis was measured as the cellular uptake of FITC-dextran (Sigma-Aldrich) and was quantified by flow cytometry. Briefly, DC (5 × 10^5 ^cells/well) were incubated in medium containing FITC-dextran (0.5 mg/ml; molecular mass 40 kDa) for 2 h at 37°C. As a negative control, DC were precooled to 4°C prior to incubation with FITC-dextran at 4°C for 2 hours. Subsequently, incubated cells were washed 3 times with cold PBS and analyzed by FACS.

### Cytokine and chemokine ELISA

Cytokine production was determined by sandwich ELISA. DC were cultured in 12-well culture plates (1 × 10^6^cells/ml or 2 × 10^6 ^cells/ml) and pretreated with various concentration (0.1, 1, 10, 25, 50 μM) of DHA for 24 h, followed by LPS (0.1 μg/ml) for 12 or 24 h. Supernatants were harvested and subjected to ELISA. The detection limits were: 15 pg/ml for IL-6, TNFα, and IL-10, 30 pg/ml for IL-23 and IL-12p70, 20 pg/ml for CCL-4 and 4.7 pg/ml for IL-27.

### Real-time RT-PCR

The expression of p19, p35, p40, IL-27p28, and EBI3 was detected by SYBR Green-based real-time RT-PCR. RNA was prepared from 4 × 10^6 ^purified CD11c+ DC using an Ultraspec RNA isolation system according to the manufacturer's instructions (Biotecx Laboratories, Houston, TX). RNA (1 μg) was reversed transcribed to cDNA and subjected to real time PCR. The PCR mixture (20 μl), consists of 4 μl diluted cDNA, 16 μl of SYBR Green containing the PCR master mix and 150 nM of each primer. Real-time PCR was performed using the Stratagene Mx3005P. The following primers were used: p19 sense, 5'-TGCTGGATTGCAGAGCAGTAA-3' and antisense, 5'-ATGCAGAGATTCCGAGAGA-3'; p35 sense, 5'-GAGGACTTGAA GATGTACAG-3' and antisense, 5'-TTCTATCTGTGTGAGGAGGGC-3'; p40 sense, 5'-GACCCTGCCGATTGAACTGGC-3' and antisense, 5'-CAACGTTGCATCCTAGGA TCG-3'; p28 sense, 5'-TCTGGTACAAGCTGGTTCCTGG-3' and antisense, 5'-TAGCCCTGAACCTCAGAGAGCA-3'; EBI3 sense, 5'-GAGGGTCCGGCTTGATGAT T-3' and antisense, 5'-CACGGTGCCCTACATGCTAA-3'; β-actin sense, 5'-TCCACCACCACAGCTGAGAGG-3' and antisense, 5'-CAGCTTCTC TTTGATGTCACG-3'. The cycling conditions were 95°C for 15 s, 75°C for 1 min, 57°C for 30 sec, for 40 cycles, followed by a melting point determination or dissociation curves. The expression level of each gene is indicated by the number of cycles needed for the cDNA amplification to reach a threshold. The amount of DNA is calculated from the number of cycles by using standard curves and the results are normalized to β-actin.

### PPARγ binding to PPRE containing oligonucleotides

Nuclear extracts were prepared as recommended by the manufacturer (Cayman Chemicals) from 5 × 10^6 ^DC treated as described in Results. The amounts of activated PPARγ were determined by using an ELISA based kit with immobilized oligonucleotides containing peroxisome proliferator responsive element (PPRE). 2 μg nuclear extract were incubated in each well overnight at 4°C without agitation. Treatment with primary Ab specific for PPARγ was followed by a secondary antibody conjugated to HRP and colorimetric readout at 450 nm. Detection antibodies and positive controls were provided with the manufacturer's kit.

### NFκB translocation assay

DC were incubated in 50 μM DHA for 24 h and treated with 0.1 μg/ml LPS for 1 h. Nuclear proteins were prepared using the nuclear extract kit (Active Motif) according to the manufacturer's protocol. The amount of active NFκBp65 in the nuclear extract was detected using ELISA based TransAM NFκB kits containing immobilized NFκBp65 consensus site oligonucleotides. 5 μg nuclear extract was added to each well, followed by primary antibodies that recognize a p65 epitope accessible only in activated p65 bound to target DNA. An HRP-conjugated secondary antibody provides a sensitive colorimetric readout quantified at 450 nm. Results are expressed as relative activity, *i.e*. absorbance values above those observed in samples with no LPS stimulation.

### Cell extracts and Western blots

Whole cell extracts were generated by lysing DC in buffer containing 50 mM Tris-HCl pH 7.4, 150 mM NaCl, 1 mM EDTA, 1% sodium deoxycholate, 0.1% SDS 1% Triton X-100, 1 mM PMSF, and protease inhibitor cocktail (Sigma-Aldrich). Samples were run on SDS-PAGE gels and transferred onto nitrocellulose membrane. The membrane was probed with primary antibodies against IκBα and GAPDH, followed by infrared conjugated secondary antibodies and scanned using the ODYSSEY Infrared Imaging system (LI-COR).

### In vivo experiments

4 wks old C57BL/6 mice (groups of 5) were fed with control diet (no DHA) or DHA-enriched diet (0.22% DHA) (Table 1) for 5 wks. 12 h after inoculation of 50 μg LPS i.p., expression of p40, p19, p35, p28 and EBI3 was determined by qRT-PCR in purified splenic CD11c+ DC. The DHA supplement, under the trade name DHASCO, was obtained from Martek Biosciences Corporation (Columbia, MD). The diet was produced at Research Diets Incorporated (New Brunswick, NJ).

**Table 1:** Control and DHA diet analysis

	Control Diet		DHA Diet	
	**gm%**	***kcal%***	**gm%**	***kcal%***

Protein	20	20	20	20

Carbohydrate	61	59	61	59

Fat	10	22	10	22

Total		100		100

kcal/gm	4.1		4.1	

				

**Ingredient**	**gm**	***kcal***	**gm**	***kcal***

Casein, 80 Mesh	0	0	0	0

Casein, Alcohol Extracted	200	800	200	800

L-Cystine	3	12	3	12

				

Corn Starch	150	600	150	600

Maltodextrin 10	150	600	150	600

Sucrose	100	400	100	400

Dextrose	200	800	200	800

				

Cellulose, BW200	50	0	50	0

				

Soybean Oil	0	0	0	0

Safflower Oil, High Oleic	100	900	94.5	850.5

DHASCO (40% DHA)	0	0	5.5	49.5

t-BHQ	0.014	0	0.014	0

Mineral Mix S10022G	35	0	35	0

				

Vitamin Mix V10037	10	40	10	40

Choline Bitartrate	2.5	0	2.5	0

				

**Total**	**1000.514**	***4152***	**1000.514**	***4152***

Total composition of experimental diets per manufacturer's analysis

### Purification of splenic CD11c+ DC

Spleens cut into small pieces were incubated in HBSS w/Ca^+2^, Mg^+2 ^containing 0.5 mg/ml Liberase TL and 1 mg/ml DNase I at 37°C for 30 min. Debris was removed by filtering cells through cell strainers (70 μm). Spleen CD11c+ DC were purified using the autoMACS system and CD11c magnetic beads as recommended by the manufacturer (Miltenyi Biotec).

### Statistical analysis

Results are described as mean +/- SD. Comparisons between two groups were done using Student *t *test, whereas comparisons among multiple groups were done by one way ANOVA. Statistical significance was determined as *p *values less than 0.05.

## Results

### DHA maintains an immature phenotype in DC

**Figure 1:**
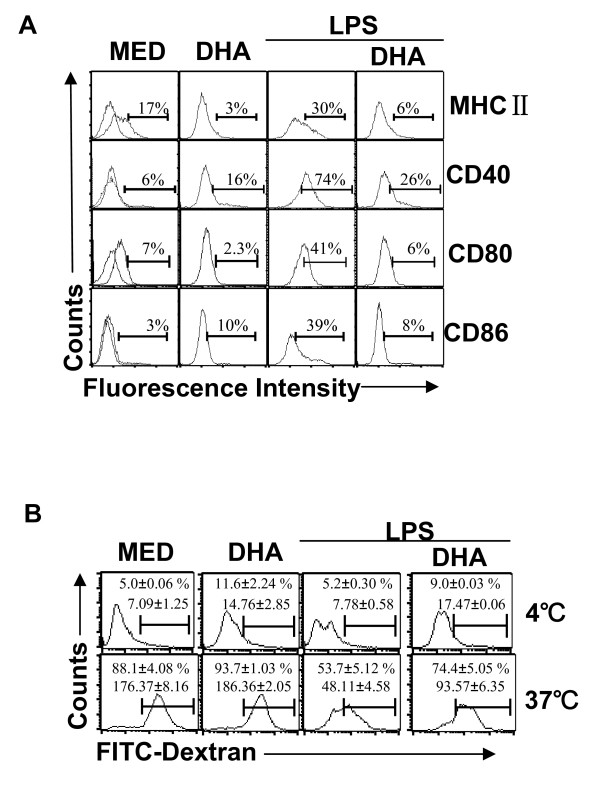
**DHA maintains DC immature phenotype and high endocytic capacity**. CD11c+ DC (1 × 10^6^/ml) were treated with 50 μM DHA for 24 h, followed by LPS (0.1 μg/ml) for another 24 h. (**A**) Cells were analyzed for MHCII, CD40, CD80 and CD86 expression by flow cytometry. One representative experiment of three is shown. (**B**) DC were treated as in (A) and 5 × 10^5 ^cells were incubated with FITC-dextran (0.5 mg/ml) at 4°C (negative control) or 37°C and assessed for endocytosis by flow cytometry. Results are expressed as percentage positive cells and geometric mean fluorescence (GMF) +/- SD.

Increased expression of MHCII and costimulatory molecules are hallmarks of DC maturation. To determine whether DHA affects DC maturation, CD11c+ bone marrow-derived DC were pretreated with DHA for 24 h followed by LPS for an additional 24 h. Immature DC displayed low levels of MHCII, CD40, CD80, and CD86, and expression was dramatically increased following LPS stimulation. DHA prevented the LPS-induced upregulation of MHCII and costimulatory molecules (Fig. 1A).

In contrast to immature DC which take up antigens efficiently, antigen uptake is significantly reduced in mature DC. We investigated the effects of DHA on the capacity of immature and LPS-matured DC to take up FITC-dextran, and found that DHA prevented the decrease in endocytosis following LPS treatment (Fig. 1B). Taken together, these results suggest that DHA contributes to the maintenance of an immature status in LPS-stimulated DC.

### DHA prevents cytokine and chemokine release from LPS-stimulated DC

**Figure 2:**
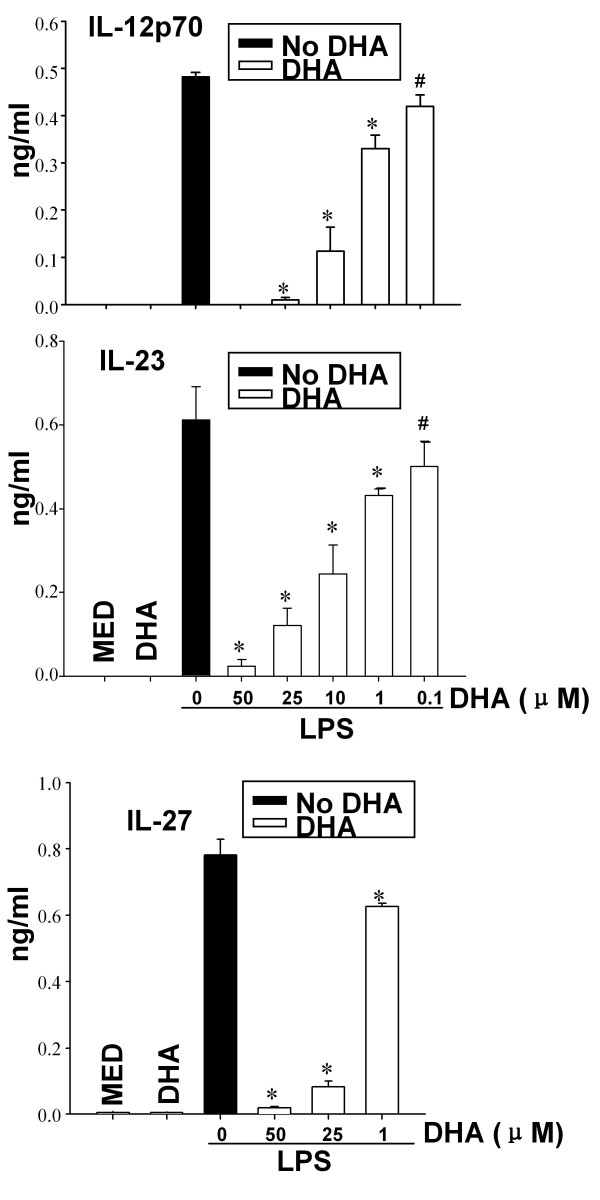
**DHA prevents expression of IL-12 family cytokines in LPS-treated DC**. DC (1 × 10^6^/ml for IL-12p70 and IL-27 and 2 × 10^6^/ml for IL-23) were cultured in the presence of different concentration of DHA for 24 h, followed by LPS for 12 h (for IL-23) or 24 h (for IL-12p70 and IL-27). Supernatants were subjected to ELISA. Data represent the mean +/- SD. * *p *< 0.01, #*p *< 0.05, compared with LPS-treated samples (no DHA).

**Figure 3:**
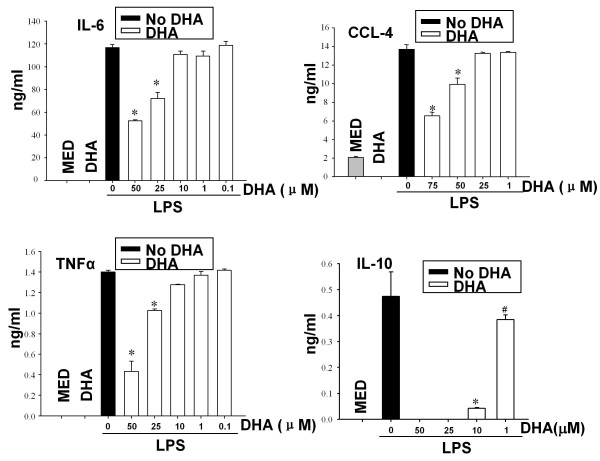
**DHA prevents cytokine production in LPS-treated DC**. CD11c+ DC (2 × 10^6^/ml for IL-10 or 1 × 10^6^/ml for TNFα, IL-6 and CCL-4) were treated with various concentrations of DHA for 24 h followed by LPS (0.1 μg/ml) for an additional 24 h. Supernatants were harvested and assayed for IL-6, CCL-4, IL-10 and TNFα by ELISA. Two independent cultures were tested in triplicate. Results are expressed as mean +/- SD. * *p *< 0.01, #*p *< 0.05, compared to LPS-treated samples (no DHA).

In contrast to immature DC, LPS-treated DC secrete proinflammatory cytokines and chemokines including IL-12p70, IL-23, IL-6, TNFα, CCL3 and CCL4, and anti-inflammatory cytokines such as IL-10. To investigate the effect of DHA on cytokine/chemokine production, we pretreated DC with DHA, followed by LPS stimulation. DHA inhibited in a dose-dependent manner the release of the IL-12 cytokine family, *i.e*. IL-12p70, IL-23 and IL-27 (Fig. 2). We observed a similar DHA inhibition of IL-6, TNFα, CCL-4, and IL-10 secretion in LPS-stimulated DC (Fig. 3).

**Figure 4:**
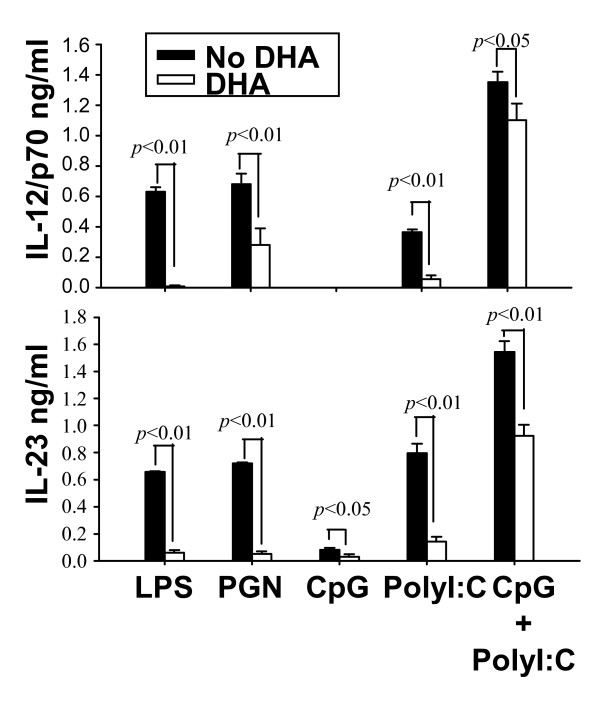
**Effects of DHA on IL-12p70 production initiated by signaling through various TLRs**. CD11c^+ ^DC (1 × 10^6 ^cells/ml for IL-12p70 and 2 × 10^6 ^cells/ml for IL-23) were pretreated with 25 μM DHA for 24 h followed by different TLR ligands (LPS 0.1 μg/ml, PGN 10 μg/ml, CpG 1 μM, poly I:C 50 μg/ml, CpG 1 μM + poly I:C 50 μg/ml) for an additional 12 h (for IL-23) or 24 h (for IL-12p70). Supernatants were harvested and assayed for IL-12p70 or IL-23 by ELISA.

To investigate whether DHA could affect cytokine production in response to signaling through various TLRs, we pretreated DC with DHA, followed by stimulation with LPS (TLR4 ligand), PGN (TLR2 ligand), CpG (TLR9 ligand), poly I:C (TLR3 ligand) and combined CpG and poly I:C treatment. We observed profound inhibition of both IL-12p70 and IL-23 for LPS, PGN and poly I:C stimulation, and reduction in cytokine production for the combined CpG and poly I:C treatment (Fig. 4).

### DHA prevents IL-12p70 production through effects on PPARγ and NFκB

In an effort to elucidate the transcriptional mechanisms we focused on the peroxisome proliferator activated receptor γ (PPARγ), which has the capacity to bind unsaturated fatty acids including DHA, and on the NFκB signaling pathway.

**Figure 5:**
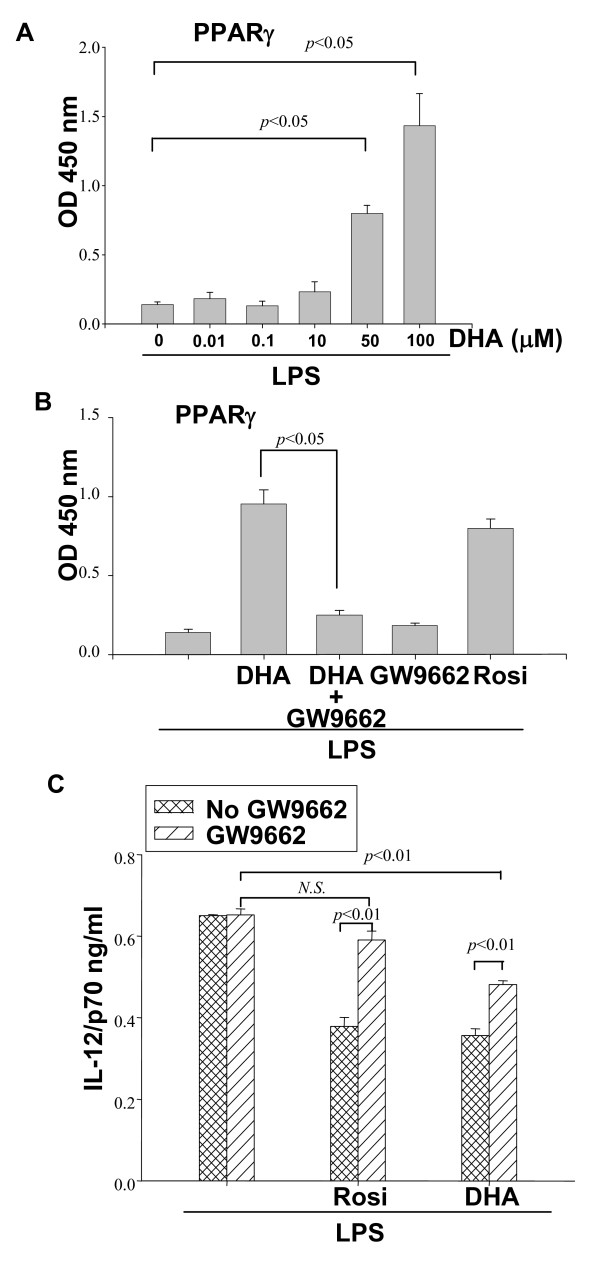
**DHA activates PPARγ**. **(A) **CD11c+ DC (5 × 10^6^) were pretreated with increasing concentrations of DHA for 24 h, followed by 0.1 μg/ml LPS stimulation for 16 h. Activation of nuclear PPARγ was evaluated through binding to oligonucleotides containing PPRE as described in Methods. (**B**) DC were pretreated with the PPARγ inhibitor GW9662 (10 μM) for 2 h, followed by 50 μM DHA or 1 μM of the specific PPARγ agonist Rosiglitazone (Rosi) for 24 h, and LPS (0.1 μg/ml) for an additional 16 h. Activation of PPARγ was determined as described in (A). **(C) **CD11c+ DC (1 × 10^6^/ml) were pretreated with 4 μM GW9662 for 2 h before the addition of 5 μM DHA or 0.4 μM Rosi (for 24 h) and LPS for an additional 24 h. Supernatants were collected and IL-12p70 production was determined by ELISA.

DC were pretreated with increasing concentrations of DHA for 24 h, followed by LPS stimulation. Nuclear extracts were tested for PPARγ activation by using a PPARγ regulatory sequence (PPRE)-containing oligonucleotide binding assay. We observed high levels of PPARγ binding in DC treated with DHA concentrations that were inhibitory for cytokine production (Fig. 5A). The specificity of PPARγ binding in DC treated with DHA and LPS was confirmed by use of the specific PPARγ inhibitor GW9662, which reduced binding to control levels. As a control for PPARγ binding specificity, we used the PPARγ agonist Rosi which induced binding in DC treated with LPS in the absence of DHA (Fig. 5B).

Next, we assessed the involvement of PPARγ in the DHA-mediated inhibition of cytokine production. The PPARγ inhibitor GW9662 did not affect LPS-induced IL-12p70 production (Fig. 5C). PPARγ activation by Rosi prior to LPS treatment resulted in approximately 40% reduction in IL-12p70 production. As expected, this reduction was abolished by the PPARγ inhibitor GW9662 (Fig. 5C). Similar to Rosi, DHA pretreatment resulted in a reduction in IL-12p70. However, in this case, the reduction was only partially reversed by GW9662 (Fig. 5C), an indication that other factors in addition to PPARγ mediate the inhibitory effect of DHA on IL-12p70 production.

**Figure 6:**
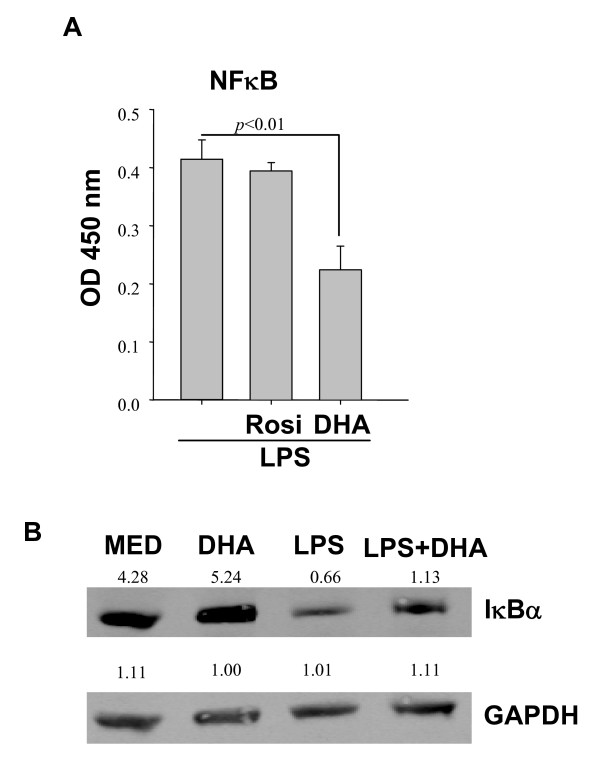
**DHA blocks NFκB p65 nuclear translocation**. (**A**) 1 × 10^7^DC were preincubated with (50 μM) DHA or (1 μM) Rosi for 24 h, followed by LPS treatment for 1 h. Presence of NFκB p65 in DC nuclear extracts was determined by binding to κB containing oligonucleotides. Results are expressed as relative activity, *i.e*. absorbance values above those observed in the absence of LPS stimulation. (**B**) DC were pretreated with DHA for 24 h, followed by LPS stimulation for 40 min, and cell lysates were subjected to Western blot analysis for IκBα. Numbers represent relative densitometric levels. One experiment out of three is shown.

Since NFκB is a major transcription factor for the expression of cytokine/chemokine genes, we evaluated the possible role of DHA on NFκB activation in LPS-treated DC. Similar to the PPARγ activation assay, we prepared DC nuclear extracts and measured p65 binding to oligonucleotides containing κB specific sequences. DC treated with LPS exhibited high nuclear κB binding which was not affected by pretreatment with Rosi (Fig. 6A), which indicates that p65 nuclear translocation in response to LPS is PPARγ independent. In contrast to Rosi, DHA reduced LPS-induced p65 binding by approximately 50% (Fig. 6A). The inhibitory effect of DHA on NFκB translocation may be mediated through the inhibition of IκB degradation. Indeed, Western blots confirmed that LPS induced a significant reduction in IκB, whereas DHA pretreated samples contained higher IκB levels (Fig. 6B).

### In vivo effect of DHA on IL-12 family cytokine expression

**Figure 7:**
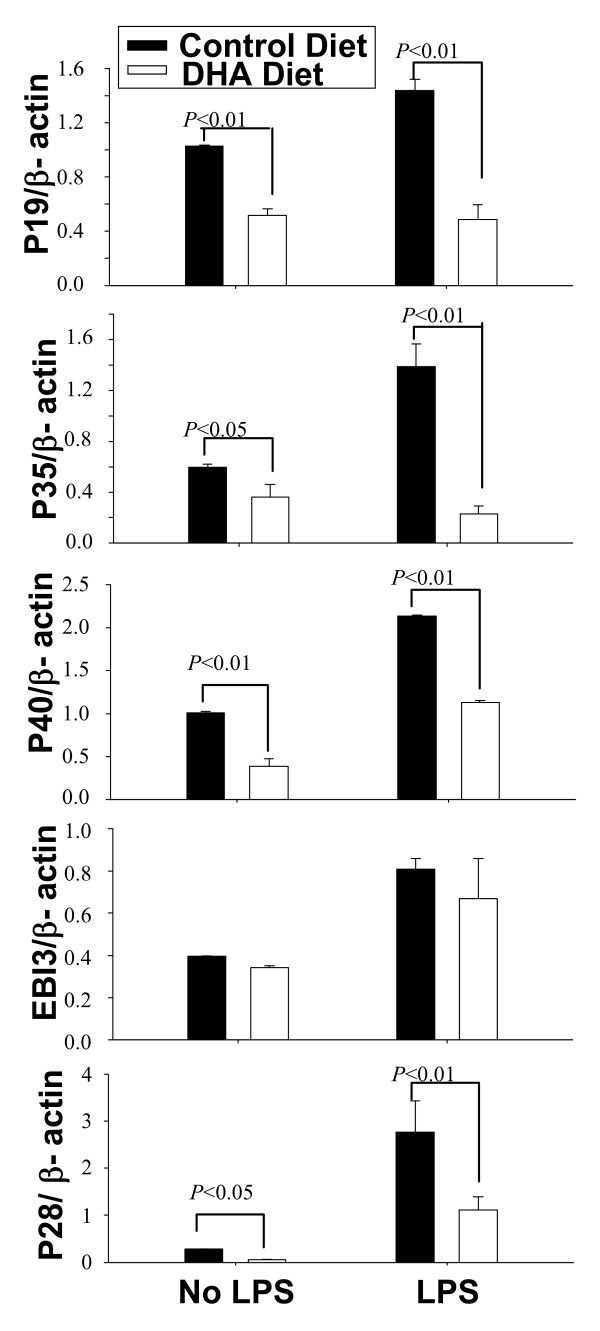
**DHA prevents in vivo expression of IL-12 family cytokines**. C57BL/6 mice were maintained for 5 wks on regular or DHA-enriched diet. Mice were injected i.p. with LPS (50 μg per mouse) and 12 h later spleen CD11c+ DC were purified. Expression of p40, p35, p19, p28 and EBI3 was determined by real time RT-PCR.

Mice were maintained on a regular or DHA-enriched diet for 5 weeks, followed by LPS inoculation. Splenic DC were purified and analyzed for expression of IL-12p40, IL-12p35, IL-23p19, IL-27p28 and IL-27EBI3. With the exception of EBI3, all other IL-12 family cytokine subunits were significantly reduced in splenic DC from mice on the DHA-enriched diet (Fig. 7). This indicates that, similar to our in vitro results, DHA affects cytokine expression in vivo in splenic DC.

## Discussion

The impact of diets rich in n-3 PUFAs and of DHA and EPA administration has been evaluated in clinical studies and various animal models, with protective effects described in models of colitis, sepsis, and stroke [18-24]. Confirming the protective anti-inflammatory effect of n-3 PUFAs, transgenic *fat-1 *mice expressing a gene encoding an n-3 fatty acid desaturase which enables production of n-3 from n-6 PUFAs, exhibit low NFκB activity, reduced levels of TNFα and IL-1β, and are protected from colitis [25].

The direct effects of DHA or EPA on various immune cell populations are not well studied. Initial reports indicated that DHA favors Th2 differentiation which is in agreement with its inhibitory effect on IL-12 production by DC [13-15,26,27]. In the present study we report on DHA effects on DC phenotype and function. In terms of phenotype, DC treated with DHA and LPS maintain an immature phenotype characterized by low expression of MHCII and costimulatory molecules (CD40, CD80 and CD86). From a functional point of view these phenotypic traits result in poor stimulatory capacity for T cells.

Exposure to DHA inhibited the production of proinflammatory molecules, *i.e*. IL-6, TNFα, CCL-4, and of the anti-inflammatory cytokine IL-10. This is in agreement with a report on human monocyte-derived DC generated in the presence of DHA [14]. In contrast, Loscher et al [28] reported that conjugated linoleic acid inhibited LPS-induced IL-12 in murine DC through upregulation of IL-10. Therefore, different PUFAs could act through different molecular mechanisms to attenuate the DC proinflammatory response. In agreement with several other reports [13-15,27], we found that DHA had a profound inhibitory effect on IL-12p70 production. In addition, we report here for the first time that DHA has a similar inhibitory effect on the other two members of the IL-12 family, *i.e*. IL-23 and IL-27. IL-23 inhibition could have a significant impact on T cell differentiation in terms of Th17, since IL-23 has been reported to maintain the Th17 functional phenotype [29-32].

The intracellular signaling pathways elicited by DHA and the final transcriptional targets are not known. One possible mediator is PPARγ, a nuclear receptor activated by endogenous and exogenous ligands, with multiple physiological functions, including immune regulation [33-35]. Eicosanoids and PUFAs, including DHA, are endogenous PPARγ ligands [36,37]. Highly expressed in DC, macrophages, T and B cells, PPARγ acts primarily as an anti-inflammatory agent [reviewed in [33-37]]. The model for ligand-dependent PPARγ transrepression of cytokine/chemokine gene expression in macrophages and DC includes ligand-dependent SUMOylation of PPARγ followed by binding and stabilization of corepressor complexes resulting in maintenance of active suppression of inflammatory genes [33]. We observed that DHA concentrations that were effective for IL-12 inhibition also induced PPARγ activation. The inhibitory effect of DHA on IL-12p70 production was partially reversed by the PPARγ inhibitor GW9662. Taken together, these results strongly suggest that the DHA effect on DC expression of IL-12, and presumably other cytokines as well, is mediated, at least partially, through PPARγ activation.

The partial reversal observed with the PPARγ inhibitor led us to consider the involvement of other factors beside PPARγ. Since NFκB, readily induced upon TLR signaling, is an essential transcription factor for cytokine/chemokine gene expression, we assessed the effect of DHA on NFκB activation. Our results indicate a reduction of approximately 50% in the amounts of nuclear p65 in DC treated with DHA and LPS, and the reduction in p65 nuclear translocation correlates with DHA-mediated IκB stabilization.

Chronic activation of immune cells, including DC, plays an essential role in inflammatory/autoimmune diseases. In contrast to extensive knowledge regarding the activation of the immune system, less is known about endogenous and exogenous factors which contribute to immune deactivation or return to homeostatic levels. Recently, the n-3 unsaturated fatty acids emerged as significant agents in resolving inflammation. Understanding their actions on various immune cells will contribute to a large degree to the future development of n-3 fatty acids and/or their derivatives as potent anti-inflammatory therapeutic agents in the prevention and treatment of autoimmune/inflammatory conditions.

## Conclusions

In this study, exposure of bone marrow-derived DC to DHA resulted in the maintenance of an immature phenotype and drastic reduction in proinflammatory cytokine release, particularly of the IL-12 cytokine family. The effect of DHA on IL-12 expression was mediated through activation of PPARγ and inhibition of NFκB. Inhibition of IL-12 and IL-23 expression was also evident in splenic DC from mice fed a DHA-enriched diet, suggesting that dietary DHA acts as an anti-inflammatory agent in vivo.

## Abbreviations

AA: arachidonic acid; COX: cycloxygenase; CpG: cytosine linked to a guanine by a phosphate bond; DC: dendritic cells; DHA: docosahexaeoic acid; EBI3: Epstein-Barr virus induced gene 3; EPA: eicosapentaenoic acid; LOX: lipoxygenase; PGN: proteoglycan; PolyI:C: Polyinosinic-Polycytidylic acid; PPARγ: peroxisome proliferator activated receptor gamma; PPRE: peroxisome proliferator responsive element; PUFA: polyunsaturated fatty acids; Rosi: Rosiglitazone; SUMO: small ubiquitin-like modifier; TMB: tetramethylbenzidine.

## Competing interests

The authors declare that they have no competing interests.

## Authors' contributions

WK, JHY and DG had substantial contributions to conception, design, interpretation of data and writing of the manuscript. WK, JHY, SA, MGT, EV carried out various experimental procedures. All authors read and approved the final manuscript.
